# Methods for Evaluating Testicular Function in Domestic Cats

**DOI:** 10.1111/rda.70042

**Published:** 2025-03-08

**Authors:** Stepanka Homola, Sait Sendag, Davut Koca, Axel Wehrend

**Affiliations:** ^1^ Veterinary Clinic for Reproductive Medicine and Neonatology Justus‐Liebig‐University Giessen Germany; ^2^ Department of Obstetrics and Gynecology Faculty of Veterinary Medicine, Van Yuzuncu Yil University Van Turkey

**Keywords:** feline, histology, sonomorphology, testicle, testicular volume

## Abstract

This study aimed to describe the physiological sonomorphology of the cat testicle and to verify the findings by histological and endocrinological analyses. Furthermore, two methods of testicle measurement and volume calculation were compared. For the study, a clinical examination of the testicles was carried out in 39 cats. The testicles were measured with the Podany testimeter and using a sonographic examination. This was followed by a castration and histological examination of the testicle. The testosterone level was measured from a blood sample. The cat testicles showed a characteristic sonomorphology. The parenchyma was homogeneous, with a distinct echogenic mediastinum. The histological examination verified that there was spermatogenesis activity in testicular tissue. It was found that the testosterone concentrations varied greatly between the animals (0.1–4.40 ng/mL) although spermatogenesis was detected in all cats. Significant correlations were detected between right and left testicular volumes of the cats according to the measured values of sonography and Podany testimeter (*p* < 0.001). In addition, there was a significant and positive correlation between testosterone and testicular volume. Testosterone levels increase with increasing testicular volume according to the sonographic method (*p* < 0.05). With the help of the sonographic measurement, significantly higher testicular volumes are calculated than were compared with the measurement method with the Podany testimeter (*p* < 0.05). The present study is the first to provide detailed information and reliable data for the evaluation of the testicle size and volume in male cats as well as for sonomorphology, which can be used as comparison values for the andrological examination of this animal species.

## 1 Introduction

Currently, scientific data on male domestic cat (*Feline catus*) reproduction is limited. In the male feline reproductive system, testicular spermatogenesis plays an important role in fertility maintenance (França and Godinho 2003). Therefore, comprehensive examination protocols of the testicle in cats are critical for andrological clinical evaluation. Testicular ultrasound examination is a widely used method in andrological examination (Davidson and Baker 2009; De Brito et al. 2015; Lapuente et al. 2020; Lürssen and Janthur 2007; Paltiel et al. 2002). In order to determine the physiological sonomorphology, it is necessary to compare the sonographic examination with a histological and functional examination of the imaged organ. To the best of our knowledge, this has not yet been done for the cat testicle. Testicle size is an important parameter in andrology, as it is associated with sperm production capacity. The measurement of testicular volume in andrology is a common method in humans and other animal species. There are various methods for testicular measurement, for example, using the Podany testimeter, the Prader testimeter (Sakamoto et al. 2007), the Hynie testimeter (Behre et al. 1989), the Rochester testimeter (Paltiel et al. 2002), the calliper (Pricking et al. 2017), and ultrasound (De Brito et al. 2015). However, the lack of standardised examination and comparative measurement protocols for testicles in domestic cats is remarkable. The aim of the study was therefore to investigate and describe the sonomorphology of the physiological feline testicles and to verify the findings by histological and endocrinological analyses. Furthermore, two methods of testicular measurement and volume calculation should be compared. Comparative studies of these methods are not available for the cat. The results will form the basis for andrological examinations of this species in the future.

## 2 Material and Methods

### 2.1 Animals

Thirty‐nine male cats of the genus Felis were available for the study. There were 33 European shorthair males, five Persian males and one Bengal male. All of the cats came from the patient population of the Clinic for Reproductive Medicine and Neonatology at Justus‐Liebig University Gießen, where they were presented for castration. The body weight of the animals varied from 1.98 to 4.39 kg. The examinations were carried out as part of the preoperative control of the animals or on the testicles removed after castration. The use of the testicles for scientific purposes was authorised by the Gießen Regional Authority (kTV 7‐2017). The inclusion criterion was that the cats had an undisturbed general condition and no pathological abnormalities in the clinical andrological examination.

### 2.2 Study Design

#### 2.2.1 General Examination

After collecting the medical history, a general examination was carried out. The examination included recording the general condition, vital parameters (heart rate, respiratory rate and temperature), assessment of the mucous membranes of the mouth and capillary refilling time, auscultation of the lungs and heart, and palpation of the abdomen and lymph nodes. All cats were weighed using precision scales to ensure an optimal anaesthetic dosage.

#### 2.2.2 Clinical Examination of the Testicles

The testicles were examined adspectorily (visual inspection) and palpatorily. During the adspection, attention was paid to symmetry, size, possible redness and an increase in circumference. In addition to checking the presence of both testicles, palpation recorded warmth, displaceability of the skin, consistency and the ability to distinguish the testicles from the epididymis. Palpation was used to subjectively determine the consistency of the testicles. Before testicular measurements with the Podany testimeter and testicular ultrasonography, intramuscular anaesthesia with ursotamine (0.1 mL per kg ketamine hydrochloride, Ursotamine 100 mg/mL, Serumwerk AG, Bernburg, Germany) and xylazine (0.1 mL per kg, xylazine hydrochloride, Xylazine 2% Bernburg, Bernburg AG, Bernburg, Germany) was performed.

#### 2.2.3 Testicular Size Measurements With the Podany Testimeter

The measurement involved determining the testicular length, width and depth of both testicles, including the skin and testicular capsules. To measure the length of the testicles, the distance from the head of the epididymis over the testicles to the tail of the epididymis was measured. The measurement was taken in the middle of the longitudinal axis. The testicular width was measured in the middle of the testicle in relation to the transverse axis. Testicular depth was also measured from the middle of the transverse axis to the longitudinal axis. Testicular length, width and depth were measured in cm. The scrotal circumference was measured using a measuring tape. To measure the scrotal circumference, both testicles were measured together in the scrotum and recorded in cm.

#### 2.2.4 Testicular Ultrasonography

**FIGURE 1:**
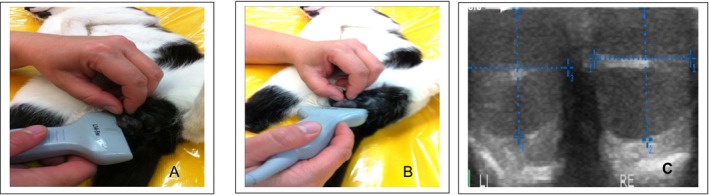
Position a multifrequency linear transducer (L 14‐5 w) longitudinally on the testicle from the lateral side (A). Application of a multifrequency linear transducer (L 14‐5 w) from the underside of the testicle (B). Ultrasound image of both testicles of a cat (transverse direction with the axes marked) (C).

The Zonare z.one ultra (Zonare Medical Systems, Erlangen, Germany) was used for sonographic visualisation of the testicles. Two transducers were used for the examinations. These were the multifrequency sector transducer (C 9‐3) and the multifrequency linear transducer (L 14‐5w). For the sonographic examination, the anaesthetised cats were placed in the left and then in the right lateral position. The testicles were sonographically examined and measured in the longitudinal‐transverse axis and in the sagittal direction (Figure 1A,B). The transverse axis was examined both dorsally and ventrally. The testicles were also examined sonographically in the same way on the longitudinal axis, the transverse axis (from dorsal to ventral), and in the sagittal direction. In addition to the measurement, the texture, echogenicity and homogeneity of the testicular tissue were assessed (Figure 1C).

#### 2.2.5 Calculation of the Testicular Volume

The testicular volume was calculated using the formula *V* = $\frac{4}{3}\frac{\pi *1}{2}\frac{a*1}{2}\frac{b*1}{2}c$. The parameters *a*, *b* and *c* are the length, width and depth of the testicles. This formula was used to calculate testicular volume in rabbits (Schüddemage et al. 2000) and boars (Young and King 1986). This calculation method was applied both to the parameters obtained by sonographic measurement and to those obtained by measurement with the podany testimeter (Sakamoto et al. 2007).

#### 2.2.6 Operative Removal of Testicles (Castration)

Anaesthesia was continued as inhalation anaesthesia with isoflurane (100 mL/100 mL, Isoflo, Albrecht, Germany) and a nitrous oxide/oxygen mixture (1:2). The cats were placed in the dorsal position, and both hind limbs were extended cranially. Tolfenamic acid (Tolfedine 4%, 4 mg/kg, Vetquinol SA, Ravensburg, Germany) was used as an analgesic. Veracin–compositum (0.5 mL/5 kg, 1 mL contains 80,000 IU (76.19 mg) benzathine—benzylpenicillin and 120,000 IU (120 mg) benzylpenicillin—procaine and 200,000 IU (258 mg) dihydrostreptomycin sulphate and 20 mg procaine hydrochloride) was used as an antibiotic. Veracin–compositum (0.5 mL/5 kg, 1 mL contains 80,000 IU (76.19 mg) benzathine‐benzylpenicillin and 120,000 IU (120 mg) benzylpenicillin—procaine and 200,000 IU (258 mg) dihydrostreptomycin sulphate and 20 mg procaine hydrochloride) was used as an antibiotic. After removing the testicles, the edges of the scrotal wound were carefully pressed together. The scrotum did not need to be sutured due to the fact that the skin sticks together very quickly. The animals were then monitored in a cat cage under the red light lamp in the recovery room and taken to the ward after waking up.

#### 2.2.7 Histological Examinations

The testicles were weighed using a precision balance and assessed macroscopically. They were then placed in a plastic tube containing 0.1 M sodium phosphate buffer (pH 7.2). The plastic tubes with the testicles were stored in the refrigerator at a temperature of around 6°C. The tunica vaginalis was removed from the testicles in the laboratory. The epididymal tail was carefully incised with a scalpel blade, and the seminal fluid was applied to a microscope slide. Subsequently, the presence of sperm in the smear was examined under a light microscope. The epididymis was then dissected free from the testicles; the testicles were cut crosswise in the centre and placed separately in labelled boxes in a jar of formol. At the Institute of Veterinary Anatomy, Histology and Embryology at Justus‐Liebig University Giessen, samples were embedded in paraffin using the Microtom embedding machine (Leica EG 1160, Leica Biosystem GmbH, Nussloch, Germany). Histological sections were prepared using a rotary microtome (Leica RM 2125 RT with disposable Leica blades). Hämatoxylin‐Eosin was also used for staining the tissue sections.

#### 2.2.8 Light Microscopic Analysis

During the histological examination of the tissue sections, the testicular structure, the staining, and the visibility of the typical tissue structures of the organ were checked, and the presence of sperm was examined. The examination was carried out using a light microscope (DMR, Leica, Wetzlar), which was connected to a digital camera (Mavica, MCV‐FD95, Sony, Cologne) with an image analysis program (Leica Image Manager, Leica, Wetzlar). This made it possible to transfer the images to a computer (GX 240, Dell, Frankfurt/M) at the same time. The images were then documented and saved on this computer.

#### 2.2.9 Collection of the Blood Sample

Blood samples were taken as part of the preoperative laboratory diagnostic screening. Blood samples were taken from all cats between 9 and 11 AM. Venous blood was sampled from the cephalic vein of the left forelimb using a sterile 20 G disposable cannula into the lithium heparin tube (4.5 mL). The processed blood samples were then frozen at −18°C until the testosterone plasma concentration was determined. The testosterone plasma concentrations were determined in the endocrinological laboratory of the Clinic for Reproductive Medicine and Neonatology, Justus‐Liebig University, Gießen.

#### 2.2.10 Testosterone Measurements

The testosterone concentration was measured using a radioimmunoassay (RIA). The hormone was determined using the methods established and evaluated by Hoffmann and Landeck (1999). The lower limit of detectability is 0.35 nmol/L or 0.1 ng/mL. The results of the testosterone determination were given in ng/ml.

#### 2.2.11 Statistical Analysis

All statistical analysis was done using Minitab (v21.4.0; LLC, Pennsylvania, USA). Descriptive statistical data was calculated for better interpretation of the results. Due to the normal distribution of the data, parametric tests were performed. A paired *t*‐test was used to compare measurement differences between the right and left testicles. An Independent sample *t*‐test was used to compare differences in testicular volume between sonography and Podany testimeter. Pearson correlation analysis was performed to detect correlations between body weight and testicular volume, and between measurements of right and left testicular volume for sonography and Podany testimeter separately. Pearson correlation was also used to detect the correlation between total testicular volume and testosterone levels of cats for sonography and Podany testimeter. The statistical significance level was defined as *p* < 0.05.

## 3 Results

### 3.1 Sonogram of the Physiological Testicle

**FIGURE 2:**
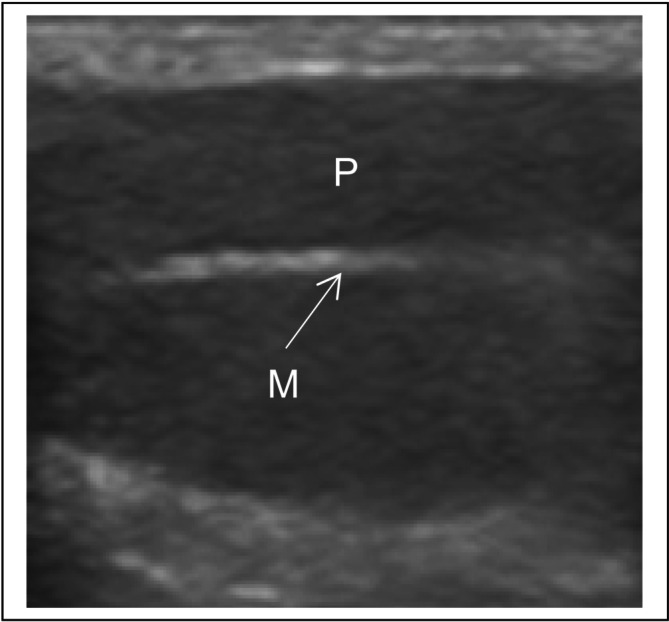
Sonogram of the physiological testicle in a male cat (P: Testicular parenchyma, M: Testicular mediastinum).

The parenchyma of the testicle is homogeneous, with a clearly echogenic mediastinum (Figure 2). A mediastinum testis was always detectable. The head, body and tail parts of the epididymis can be identified. The epididymis was typically less echogenic than the testicular parenchyma.

### 3.2 Sonographic Measurements of the Testicles

Paired *t*‐test results showed that testicle length was higher in the left testicle compared to the right testicle (*p* < 0.05). However, testicle width and depth did not change between the right and left testicle (*p* > 0.05). The measurement results are summarised in Table 1.

**TABLE 1:** Sonographically measured testicular dimensions in cats and comparison of right and left testicle measurements.

Variable	*n*	Mean	SE mean	St dev	Min	Q1	Median	Q3	Max	*p*
Length (cm)										
Right testicle	39	1.31	0.03	0.24	0.70	1.20	1.30	1.50	1.70	0.04*
Left testicle	39	1.36	0.03	0.24	0.80	1.20	1.40	1.50	1.70	
Width (cm)										
Right testicle	39	1.15	0.02	0.17	0.70	1.00	1.20	1.30	1.50	> 0.05
Left testicle	39	1.15	0.02	0.17	0.70	1.10	1.20	1.30	1.40	
Depth (cm)										
Right testicle	39	0.96	0.03	0.21	0.50	0.80	1.00	1.10	1.40	> 0.05
Left testicle	39	0.97	0.03	0.20	0.40	0.80	1.00	1.10	1.40	

* *p* < 0.05.

### 3.3 Testicular Measurements With the Podany Testimeter

Paired *t*‐test results showed that testicle length, width and depth were not different between right and left testicle (*p* > 0.05). The measurement results are summarised in Table 2.

**TABLE 2:** Measured testicular dimensions in cats with the Podany testimeter and comparison of right and left testicle measurements.

Variable	*n*	Mean	SE mean	St dev	Minimum	Q1	Median	Q3	Maximum	*p*
Length (cm)										
Right testicle	39	1.43	0.04	0.25	0.70	1.40	1.50	1.60	2.00	> 0.05
Left testicle	39	1.44	0.04	0.25	0.70	1.40	1.50	1.50	2.00	
Width (cm)										
Right testicle	39	1.02	0.03	0.22	0.40	1.00	1.10	1.10	1.40	> 0.05
Left testicle	39	1.03	0.03	0.21	0.50	1.00	1.00	1.20	1.50	
Depth (cm)										
Right testicle	39	0.92	0.03	0.21	0.30	0.90	1.00	1.00	1.30	> 0.05
Left testicle	39	0.90	0.03	0.19	0.40	0.80	0.90	1.00	1.30	

### 3.4 Correlation Between the Left and Right Testicular Volumes as Determined by Sonography and the Podany Testimeter

**FIGURE 3:**
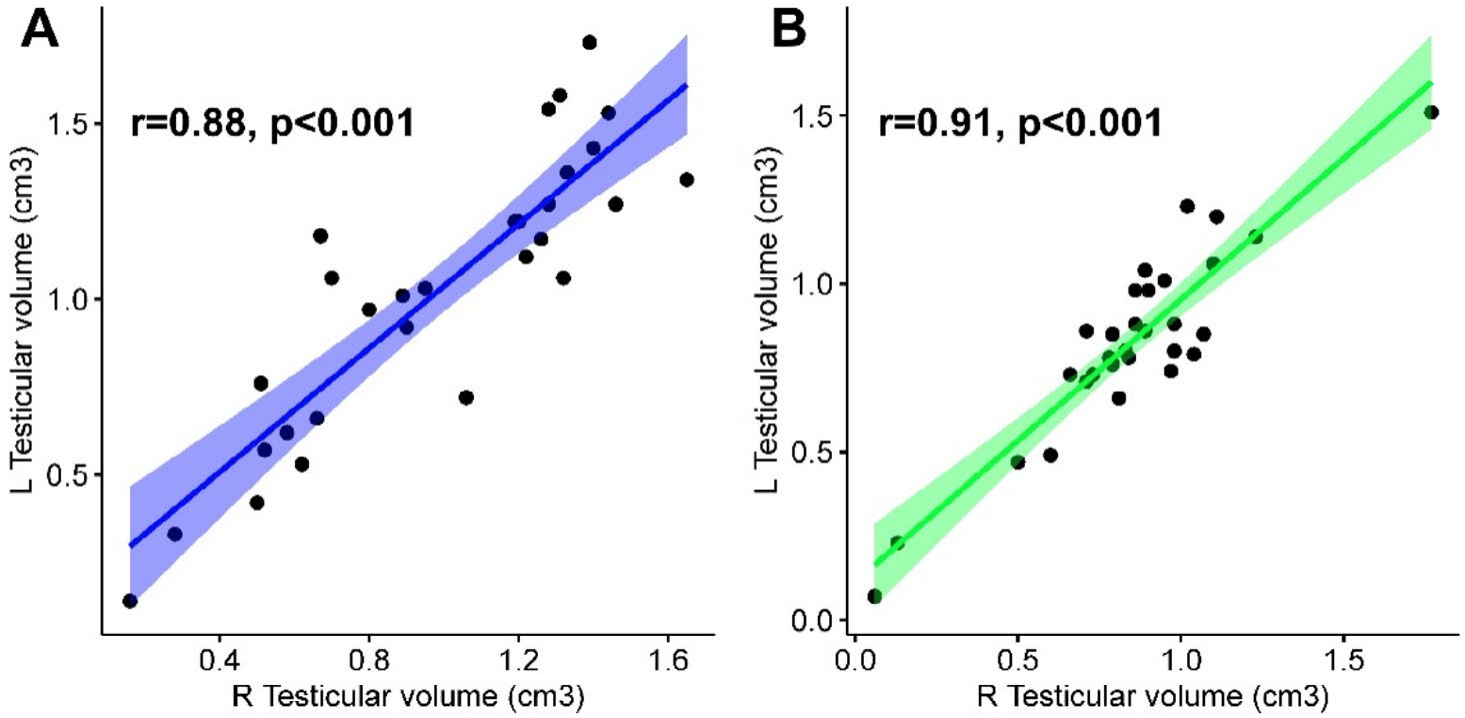
Scatter plots showing correlations between the right (R) and left (L) testicular volumes (cm^3^) of the cats according to the measured values of the sonography (A, *r* = 0.88, *p* < 0.001) and Podany testimeter (B, *r* = 0.91, *p* < 0.001).

Testicular volumes calculated by the Podany testimeter and sonography are shown in Figure 3A,B, respectively. Positive, strong and significant correlations were detected between the right and left testicular volumes of the cats according to the measured values of the sonography (Figure 3A, *r* = 0.88, *p* < 0.001) and the Podany testimeter (Figure 3B, *r* = 0.91, *p* < 0.001) respectively.

### 3.5 Comparison of Testicular Volumes Between Sonography and Podany Testimeter

The examinations of this study show a mean testicular volume measured with the Podany testimeter of 0.77 cm^3^ for the right testicle and 0.75 cm^3^ for the left testicle. The mean testicular volume measured using ultrasound was 0.93 cm^3^ for the right testicle and 0.97 cm^3^ for the left testicle. The *t*‐test for dependent samples showed that the testicular volume calculated sonographically was larger on average than the testicular volume measured by podany. The difference between the two methods was statistically significant (*p* < 0.001).

### 3.6 Testosterone Concentrations

According to descriptive data, the testosterone levels in the cats in this study ranged from 0.1 to 4.40 ng/mL (mean 1.027 ± 0.20 ng/mL).

### 3.7 Correlation Between Body Weight, Testosterone Concentration and Testicular Volume

**FIGURE 4:**
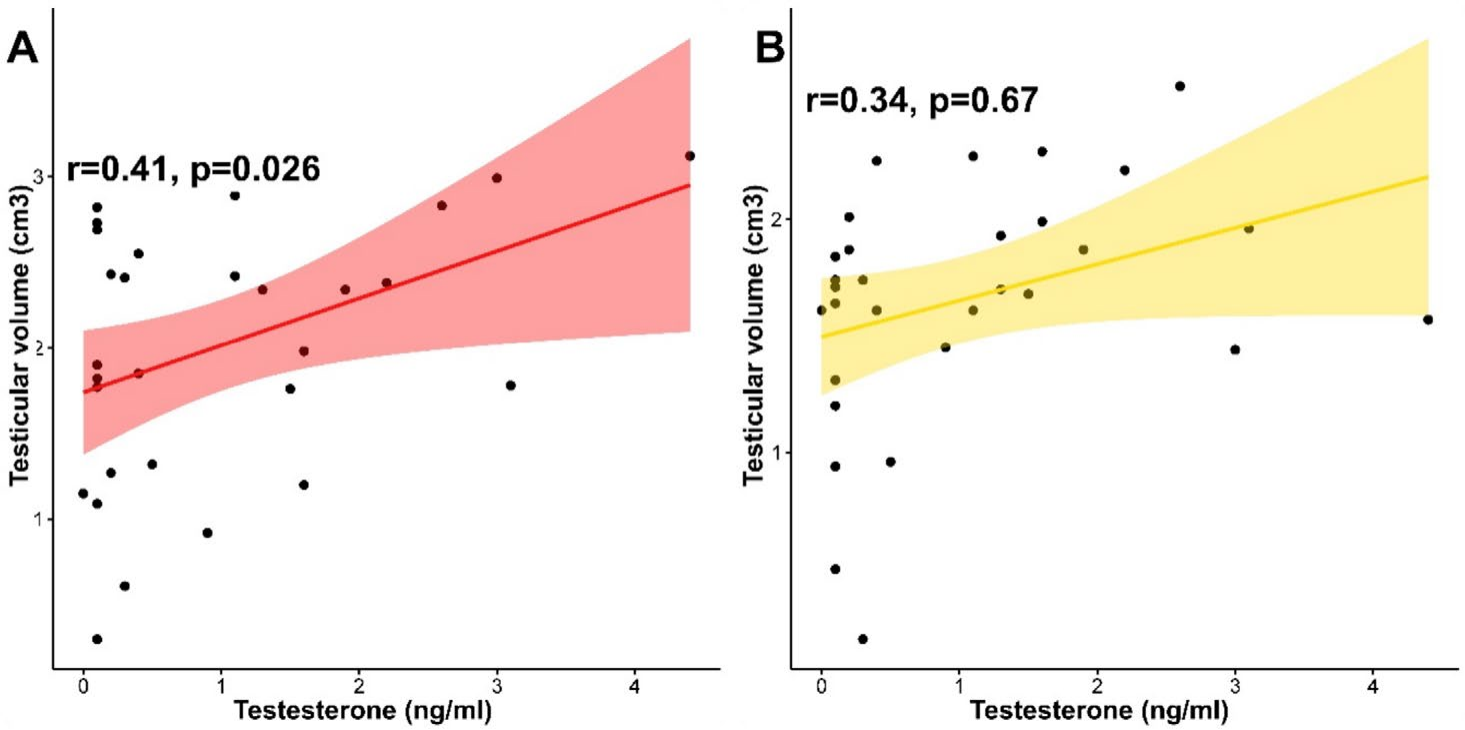
Scatter plots showing correlations between total testicular volume and testosterone levels of cats according to the sonography (Figure 4A, *r* = 0.41; *p* < 0.05) and Podany testimeter (Figure 4B, *r* = 0.34; *p* > 0.05).

A statistically significant correlation was found between body weight and testicular weight (*p* < 0.001), even if this was not particularly pronounced. A moderate, positive and significant correlation was detected between testicular volume and testosterone level according to sonography (Figure 4A, *r* = 0.41; *p* < 0.05). In addition, a weak and positive correlation between testicular volume and testosterone level was detected according to the Podany testimeter. However, this correlation was found to be not significant (Figure 4B, *r* = 0.34; *p* > 0.05).

### 3.8 Scrotal Circumference and Testicular Weights After Castration

The mean scrotal circumference of all cats was 6.34 ± 1.09 cm. Testicular weights were determined in 26 cats. The right (1.4 ± 0.6 g) and left (1.4 ± 0.6 g) testicles only differed slightly in weight. The minimum difference was 0 g, and the maximum difference was 0.3 g.

### 3.9 Histological Findings

**FIGURE 5:**
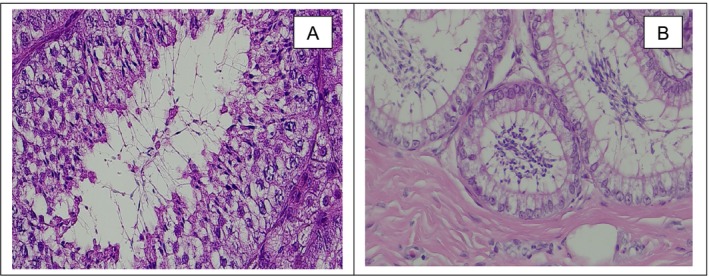
Normal histological representation of the tubulus seminiferus contortus (A) and epididymis (B) in the cat (Haematoxilin–Eosin, 400×, 50 μm). Sperm cells can be clearly seen in both histological sections.

The presence of spermatogenesis and the physiological structure of the testicular tissue (seminiferous tubules and epididymis) were investigated by the histological examination of 78 testicles. The presence of sperm was detected in the seminiferous tubules of all males. The testicular interstitium between the seminiferous tubules showed loose collagen fibres, capillaries and Leydig cells (Figure 5). The detection of sperm confirms that the animals were sexually mature.

## 4 Discussion

The aim of this study was to describe the physiological sonomorphology of the cat testicle and to verify the findings by histological and endocrinological analyses. Furthermore, two methods of testicular measurement and volume calculation should be compared. Although exact ages were not available for many animals in the present study, we can assume that all animals were sexually mature based on the histological examination and the associated detection of sperm in all testicles. Furthermore, it was remarkable that although spermatogenesis was detected in all cats, testosterone concentrations varied greatly between the animals. This observation can possibly be explained by the pronounced circadian rhythm of testosterone synthesis. The positive correlation between testicular volume and testosterone concentration was to be expected, as there are more Leydig cells in the organ with increasing testicular volume. However, this correlation only applies to healthy testicles whose mass is due to the presence of functional tissue. In our study, we observed that testosterone levels increased with increasing testicle volume (*r* = 0.41; *p* < 0.05). However, in our study, single blood samples taken from animals were evaluated for testosterone analyses. Again, there may be differences in blood testosterone values depending on the time of day. Therefore, it would be subjective to relate the testosterone values in our study directly to testicular volume, since there may be differences in repeated sampling. In cats, sonographic visualisation of the scrotum, testicles and epididymis is more difficult than in dogs due to the smaller size of the organs (Lürssen and Janthur 2007; Dzimira et al. 2017). In the present study, sonography of the feline testicle was performed. The parameters of length, width and depth were measured. The sonoarchitecture obtained corresponds to the findings from other domestic animals for physiological testicular tissue. In ultrasonography, the head, body and tail parts of the epididymis can be identified. It is noteworthy that the epididymis generally echoes less than the testicular parenchyma, as reported in some literature (Mattoon and Nyland 2015). To our knowledge, this is the first time physiological sonomorphology has been verified by histological examination. Especially when determining the normal sonoarchitecture of a tissue, it is necessary to check whether it is physiological tissue. In the present study, the testicular tissue was examined histologically for the presence of sperm to ensure that the data was obtained from healthy testicular tissue. The results of the histological examination in our study confirmed that the removed tissue belonged to a physiological testicular organ.

Testicle size is an important parameter in andrology, as it is associated with sperm production capacity. The measurement of testicular volume in andrology is a common method in humans and other animal species (Behre et al. 1989; Paltiel et al. 2002; Sakamoto et al. 2007; Pricking et al. 2017; Masoud et al. 2021, 2022). Such comprehensive and comparative studies in cats are not yet available. In a recent study using only six pairs of cat testicles, gross morphometric measurements of the testicles were performed (Kumar et al. 2023). In this study (Kumar et al. 2023), the thickness of the testicles was measured with a digital vernier calliper, the volume was calculated using the water displacement method using a measuring cylinder, and the circumference of the testicles was measured with cotton thread. It is clear from the literature presented above that there is also no comparison of methods for measuring the volume of the feline testicle and that findings from other species should not be adopted without verification.

Comparative measurements with the Podany testimeter and sonography are not yet available in the literature for the cat testicle. The current study showed that the testicular volume calculated sonographically was on average larger than the testicular volume measured by Podany (*p* < 0.001). It can therefore be concluded that the method used to obtain such measurements should not be neglected. Stremnitzer (2002) calculated the testicular volume of 34 cats based on B‐mode ultrasound measurements. The result was a mean testicular volume of the left testicle of 1.8 cm^3^ and a testicular length of 1.9 cm, and a mean testicular volume of the right testicle of 1.9 cm^3^ and a testicular length of 1.9 cm. These testicular volumes are larger than the testicular volumes in this study. The reason lies in the higher body weight of the animals by 0.4 kg in Stremnitzer (2002) and the use of a different formula. Stremnitzer (2002) used the formula of Beitz and Grote (1997), according to which *V* = 4*π*2/3. Furthermore, Stremnitzer (2002) was able to prove the dependence of the testicular volume on the season. This experiment was not carried out in our own study, as all measurements were taken in the summer. To summarise, it should be noted that ultrasound measurement produces higher values than mechanical methods for all species investigated to date. This must be taken into account when comparing different studies. This fact must also be considered when comparing individually collected patient data with reference values from the literature.

Remarkably, there is very little data on the testicular weight of sexually mature cats. Even the standard works on anatomy and reproductive medicine contain no information. An average testicular weight of 1.4 g was determined for the left and right testicles. In the study by França and Godinho (2003), the average testicular weight of the males was 1.2 g. Stremnitzer (2002) recorded 2.04 g for the left testicle and 2.11 g for the right testicle. For the practical andrological examination of a male cat, the testicular weight parameter is irrelevant as the testicles remain in the animal in breeding cats. However, for pharmacological studies on the effect of drugs designed to suppress testicular function, as an alternative to surgical castration, reference values for testicular weight in cats would be useful. The values presented here should be verified on larger numbers of animals in the future. A statistically significant correlation was found between the body weight and the weight of the testicles, even if this was not particularly pronounced. This is not surprising given that these were adult or almost adult animals.

## 5 Conclusion

The present study is the first to provide detailed testicular parameters for domestic cats, in particular the sonomorphology, which has been verified by histological and endocrinological examinations. The data collected should be used in the future as part of the andrological examination. If testicular dimensions collected on individual cats are to be compared with data from the literature, the body weight and, in particular, the methodology used to collect the dimensions must be taken into account in addition to age.

## Author Contributions

**Stepanka Homola:** conceptualisation; investigation, methodology, writing – original draft, writing – review and editing. **Sait Sendag:** conceptualisation, methodology, validation, investigation, writing – original draft, writing – review and editing. **Davut Koca:** conceptualisation; investigation, methodology, writing – original draft, writing – review and editing. **Axel Wehrend:** writing – original draft, writing – review and editing, supervision.

## Conflicts of Interest

The authors declare no conflicts of interest.

## Data Availability

The data sets generated for this study are available on reasonable request from the corresponding author.
